# Placebo effects in a RCT assessing 30 days of low dose Cannabidiol (CBD) treatment for psychological distress in stressed students at risk for depression

**DOI:** 10.1186/s42238-025-00366-9

**Published:** 2025-11-28

**Authors:** Alexander Winkler, Annelie C. Meis, Christiane Hermann

**Affiliations:** Department of Clinical Psychology and Psychotherapy, Justus-Liebig- University, Otto-Behaghel-Str. 10 F, D-35394, Giessen, Germany

**Keywords:** CBD, Cannabidiol, Placebo, Stress, Depression, Anxiety, Sleep, Psychological distress, Treatment

## Abstract

**Background:**

Cannabidiol (CBD) has been suggested to have a therapeutic role for certain mental health conditions despite a lack of empirical evidence (Khan et al. 2020; Bonaccorso et al. 2019; Kirkland et al. 2022). The aim of the RCT (DRKS00030971) was to assess the effect of a daily sublingual application of a low dose over-the-counter 10% full spectrum CBD-oil over 30 days, compared to a placebo-oil and a no-treatment control group without application of any oil on perceived stress and psychological distress in a sample of stressed students. Another goal was to assess the role of treatment expectation on the potential placebo effect.

**Methods:**

A sample of 180 highly stressed university students at risk of depression were randomized to a one-month intervention with CBD-oil or placebo-oil or a no-treatment control group. All participants received the same information about CBD. Participants took the respective oil for 30 consecutive days. Measures of psychological distress were administered 3 times (before, after intervention, 4 weeks follow-up).

**Results:**

After 30 days of intervention, groups differed significantly in decrease of stress, depressive symptoms, and somatization, but not regarding other outcomes of psychological distress (anxiety, sleep quality, increase of wellbeing). Participants in the CBD and placebo group benefitted significantly more compared to the no-treatment control group. No significant difference in any outcome was shown between the CBD-oil and the placebo-oil group. Interestingly, treatment expectation was not correlated with the decrease of symptoms in the placebo group, but with a direct rating of treatment effects at post-treatment.

**Conclusions:**

While we could not find a differential efficacy of CBD treatment on perceived stress and psychological distress, the results of the study suggest a substantial placebo response in low dose CBD treatment. Explicit treatment expectation as an underlying mechanism seems to play a minor role in explaining this placebo effect, but was correlated with the a posteriori subjective rating of treatment effects at post-treatment.

**Trial registration:**

Pre-registered on 22/12/2022 at DRKS-ID: DRKS00030971.

**Supplementary Information:**

The online version contains supplementary material available at 10.1186/s42238-025-00366-9.

## Background

Cannabidiol (CBD) has become hugely popular for (purportedly) improving mood and well-being and is most often used for this purpose despite a current lack of medical licensing for such indications (Bonaccorso et al. 2019). CBD is widely available online, in CBD stores, and even in drugstores, leading to a dramatic increase in the use of commercial CBD products, with retail sales projected to reach $16 billion by 2025. In 2018, CBD with Doses up to 25 mg/kg was approved by the FDA for the treatment of Dravet syndrome and Lennox-Gastaut syndrome (see Meissner and Cascella 2025). According to the American Society of Clinical Oncology (ASCO) guidelines, CBD-based treatments may be considered for cancer patients with refractory nausea and vomiting despite optimal antiemetic prophylaxis. CBD appears to have antipsychotic, antidepressant, anxiolytic, anti-craving and pro-cognitive effects and is also being investigated for various psychiatric conditions (Bonaccorso et al. 2019; Kirkland et al. 2022; Ricci et al. 2025; Martinotti and Di Forti 2025; Khan et al. 2020); however no FDA approvals have been granted for these indications thus far (Meissner 2005; Meissner and Cascella 2025).

While Δ⁹-tetrahydrocannabinol (THC) is the primary psychoactive constituent of cannabis, the plant contains over 100 other cannabinoids, including cannabidiol (CBD), cannabigerol (CBG), and cannabinol (CBN), each with distinct pharmacological profiles (ElSohly et al. 2016). When whole-plant cannabis is consumed, these compounds may interact synergistically or antagonistically. This phenomenon is often referred to as the “entourage effect” which potentially modifies both therapeutic and adverse effects (Russo 2011). CBD is a non-intoxicating constituent of Cannabis and is generally touted for its broad therapeutic potential, including anxiolytic, antipsychotic, analgesic, anti-inflammatory, antioxidant, and anticonvulsant effects (Crippa et al. 2018). Although CBD interacts with the endocannabinoid system, it exhibits low affinity for CB_1_ and CB_2_ receptors and is thought to exert many of its anxiolytic and antidepressant effects via non-endocannabinoid mechanisms, including activation of 5-HT_1A_ serotonin receptors and transient receptor potential vanilloid type 1 (TRPV_1_) channels (Campos and Guimarães 2008; Blessing et al. 2015; Tham et al. 2019). Notably, these effects often follow a bell-shaped dose–response curve, with therapeutic benefits observed only within a narrow dosage range (Linares et al. 2019; Zuardi et al. 2017; for a comprehensive overview of the complex pharmacodynamics of CBD, see Peng et al. (2022). However, the exact mechanisms of CBD are not yet clear, and there is still very limited evidence for clinical efficacy of CBD, except in the treatment of epilepsy (Billakota et al. 2019). While CBD has generally been well tolerated according to human studies (WHO Expert Committee on Drug Dependence 2018), potential side effects and interactions such as diarrhea, nausea, vomiting, fatigue, somnolence, and elevated liver enzymes have been reported (Huestis et al. 2019). While most acute and prolonged adverse effects are mild to moderate, some more serious adverse effects including elevated transaminases, convulsion, and upper respiratory tract infections are also reported in rare cases (Souza et al. 2022). Although there is currently insufficient high-quality evidence to recommend the clinical use of CBD for any psychiatric disorder, several reviews (Khan et al. 2020; Bonaccorso et al. 2019; Kirkland et al. 2022; Lichenstein 2022) suggest that there is growing evidence for stress-reducing (Crippa et al. 2021; Appiah-Kusi et al. 2020), anxiolytic (Bahji et al. 2020; Skelley et al. 2020; Faria et al. 2020; Bergamaschi et al. 2011; Gournay et al. 2023; Zuardi et al. 2017) and antidepressant (Pinto et al. 2020) properties of CBD. A recent meta-analysis (Bahji et al. 2020) aimed to evaluate the effectiveness and acceptability of cannabinoids (i.e. tetrahydrocannabinol, nabilone or CBD) in treating anxiety and trauma-related disorders. Among the identified 14 trials, only 3 trials evaluated a one-week intake of CBD in a total of 42 patients. The effect sizes suggest an anxiolytic effect (*g* = 0.38 [95% CI, −1.28 to 0.52]), yet were not significant.

Linares et al. (2019) aimed to investigate the acute effects of different doses of CBD compared to placebo in healthy males (*N* = 57) undergoing a simulated public speaking test (SPST). Participants received oral CBD at doses of 150 mg, 300 mg, 600 mg, or placebo in a double-blind procedure. Results showed that treatment with 300 mg of CBD significantly reduced anxiety during the speech, while no significant differences were observed with CBD doses of 150 mg, 600 mg, or placebo. These findings suggest that CBD might have anxiolytic properties in humans, with similar dose-response curves to those observed in animal studies. In a recent study, Crippa et al. (2021) investigated the efficacy of CBD in reducing emotional exhaustion and burnout symptoms in healthcare workers treating COVID-19 patients. It involved 120 participants receiving either CBD (300 mg/day) plus standard care or standard care alone for 28 days. Results showed a significant reduction in emotional exhaustion scores among CBD recipients measured on days 14, 21, and 28. The study highlights the potential benefits of CBD in alleviating burnout symptoms.

Moreover, a substantial publication bias was noted due to methodological limitations (e.g., blinding of participants, randomization issues) and the quality of evidence for primary and secondary outcomes was actually low to moderate (Bahji et al. 2020). In a review assessing the efficacy of CBD in treating mood disorders (Pinto et al. 2020), identifying 16 relevant articles. However, in most of these studies CBD was given combined with THC. In those studies evaluating CBD alone, the patients had a variety of primary diagnosis (e.g., cannabis use disorder, nicotine addiction, epilepsy, mania) or were healthy. While some studies suggested the potential benefits of CBD in alleviating depressive symptoms, the evidence lacks consistency and robustness, with no clinical trials specifically targeting mood disorders or assessing affective symptoms as primary outcomes.

Aside from very limited evidence for the efficacy of CBD in alleviating (sub)clinical levels of depressive and anxiety symptoms, little is known about the underlying pharmacological and putatively related psychological mechanisms (e.g. changes in self-efficacy) of CBD’s potential benefit. Specifically, there are no trials testing CBD against placebo. Placebo responses are largely driven by implicit and explicit expectations of treatment efficacy which can be acquired by associative learning such as classical conditioning or observational learning, are influenced by verbal information and contextual factors (Enck et al. 2013). Benedetti et al. (2011) proposed that the placebo effect can be conceptualized as a psychosocial context effect, conveying to the patient the administration of a beneficial treatment, thereby influencing their expectations regarding the treatment efficacy. The abundant media coverage, advertisements and the availability of CBD in specialized stores might serve as important contextual factors possibly contributing to CBD’s effectiveness (Wilhelm et al. 2024; Pinto et al. 2024).

For university students, elevated psychological distress (e.g., emotional suffering, stress, anxiety, depressed mood) has been reported, with prevalence rates up to 80% (Stallman 2010). Currently vulnerability-stress models of mental disorders assume that vulnerable individuals are at risk for developing psychopathological symptoms when exposed to stressful experiences. For relief of such symptoms, a growing number of people resort to medication such as antidepressants (Bergdahl & Bergdahl, 2002), benzodiazepines (McCabe, 2005) or novel psychoactive substances (Chiappini et al. 2023) as well as over-the-counter medications (Hofmeister et al., 2010; Koushede et al., 2011) like CBD, despite a lack of evidence for their effectiveness for stress reduction and their potential associated risks (e.g., unwanted side effects). Elevated psychological distress in university students predicts an increased risk of later mental disorders (Woo et al. 2024). Indeed, prevalence rates of depression are substantially higher in university students with a mean prevalence of 30.6% (Ibrahim et al. 2013) than the 9% found in the general population (Centers for Disease Control and Prevention 2010). In a study by Dickhäuser et al. (2022), perceived stress was significantly correlated with depressive symptoms (*r* =.71) in a sample of 1396 university students.

This study aimed to determine the efficacy of a CBD intervention for reducing psychological distress in a subclinical population, and to what extent this might be accounted for by placebo effects and treatment expectations. A daily sublingual application of a casual over-the-counter 10% full spectrum CBD-oil (recreational cannabis) over 30 days and a placebo oil were compared to a no-treatment control group.

Using an analog sample approach, and based on previous reports of high correlations between stress and depressive symptoms in students (Dickhäuser et al. 2022), the CBD treatment was evaluated in highly stressed university students with elevated depressive symptoms. We expected a more pronounced reduction of psychological distress in the CBD group compared to the placebo oil group and the no-treatment control group.

## Methods

We adhere to the CONSORT reporting guidelines.

### Participants

**Fig. 1:**
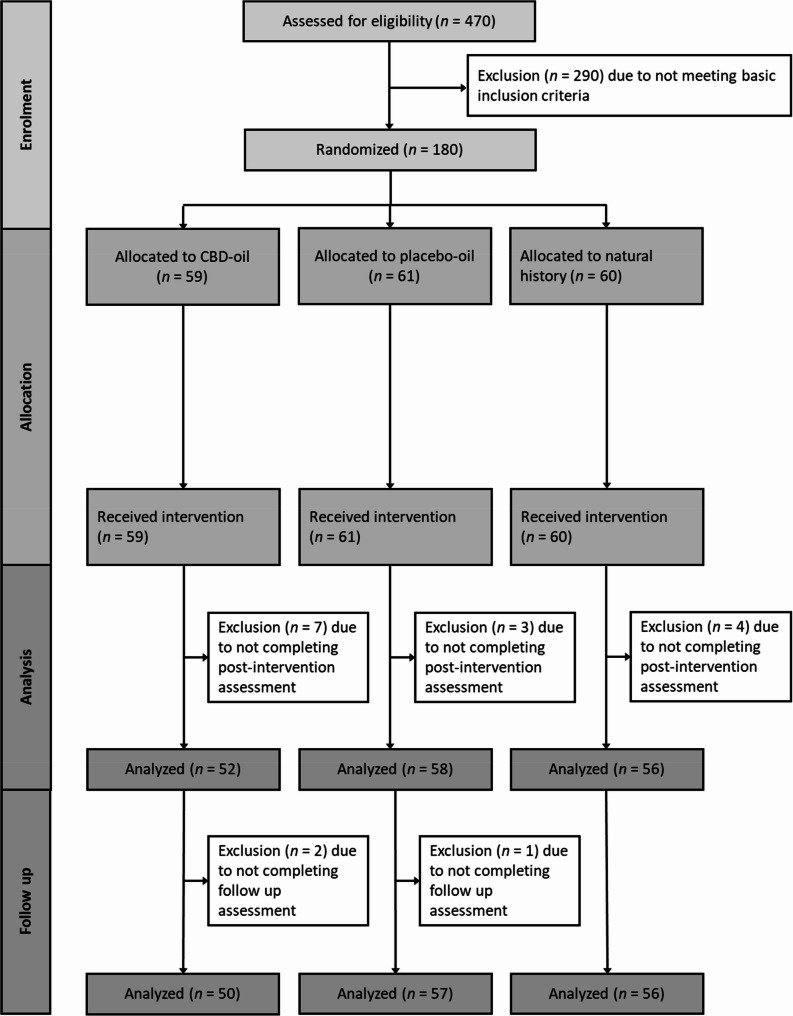

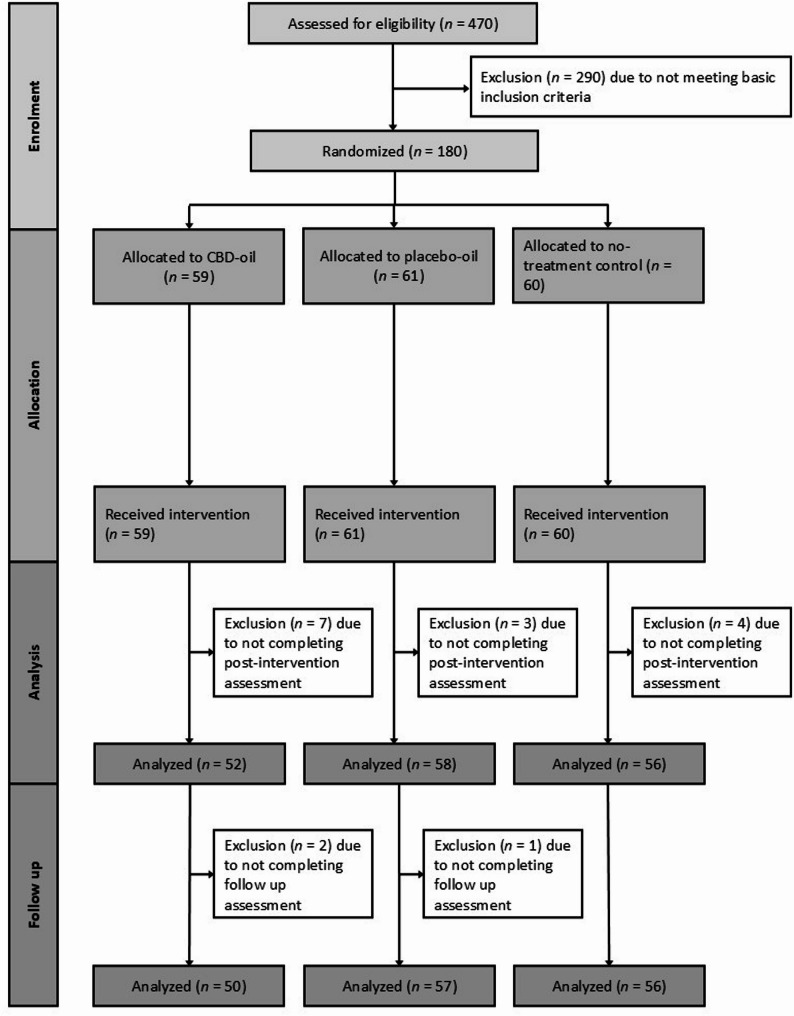
Participant flow chart

One hundred and eighty highly stressed students (81.1% females) aged between 19 and 40 years (*M* = 24.06, *SD* = 3.34) were recruited at a German university via an e-mail openly advertising a study about a CBD treatment for stress, depressive symptoms, psychological distress and sleeping difficulties. The following inclusion criteria were assessed via a phone interview: (a) age between 18 and 45 years; (b) a Perceived Stress Scale-10 (PSS-10; Klein et al. 2016) score of 23 or higher; and (c) fluency in German. In this study, we defined highly stressed university students as individuals with a PSS score higher than 1.5 standard deviation above the mean of the student subsample (*M* = 13.27, *SD* = 6.52) reported in a PSS validation study in a representative German community sample (Klein et al. 2016). Exclusion criteria were (a) allergies to any substance used in this study (full spectrum hemp extract, medium-chain triglycerides (mct) oil, hemp seed oil), (b) known/diagnosed chronic or acute physical or mental illness, (c) regular intake of any kind of medication, (d) regular use of CBD products within the last 4 weeks and (e) body weight > 85 kg (to ensure, that the content of the bottle is sufficient for the duration of the intervention). A sample size of *N* = 164 was calculated based on an a priori power analysis (G*Power 3.1.9.2) for the interaction effect of group and time, assuming a small effect size of *f* = 0.15 with a power of 90% (*α* = 0.05, *r* =.50). Assuming a dropout rate of about 10%, *N* = 180 were recruited. Fourteen participants were excluded due to not completing the post-intervention assessment (for an overview, see Fig. 1). All participants were informed about the study procedure, gave written informed consent and were reimbursed with either 30€ or another bottle of full-spectrum CBD oil (10% CBD, 10 ml) after completing participation in the study, depending on their choice. The experiment was conducted according to the Declaration of Helsinki and approved by the local ethics committee (#2022-0025). The study was pre-registered at the German Clinical Trials Register (DRKS) on 22/12/2022 (DRKS-ID: DRKS00030971; https://drks.de/search/de/trial/DRKS00030971). Participants were randomly assigned to the CBD-oil group, the placebo-oil group or the no-treatment control group. The groups were well matched and did not differ concerning age, number of females, number of study semesters, and number of stressful life events within the last week prior to baseline (see Table 1). Moreover, there were no significant differences between the placebo and CBD group regarding their adherence to oil administration as assessed by the self-reported number of days with missed oil administration. The data collection took place from 12/2022 to 11/2023.

**Table 1:** Sample characteristics and group differences at baseline

	CBD-oil (*n* = 59)	Placebo-oil (*n* = 61)	No-treatment control (*n* = 60)	Group differences
Age in years, *M (SD)*	24.25 (3.54)	23.89 (2.78)	24.05 (3.68)	*F*(2,177) = 0.18, *p* =.834
Number of females, n (%)	49 (83.1%)	48 (78.7%)	49 (81.7%)	χ²(2) = 0.07, *p* =.968
Number of study semesters, *M (SD)*	6.90 (3.64)	7.63 (4.25)	5.85 (4.10)	*F*(2,176) = 3.00, *p* =.052
Number of stressful life events within the last week, *M* (*SD*)	2.63 (1.70)	2.47 (1.70)	2.54 (1.82)	*F*(2,172) = 0.11, *p* =.893
Treatment expectation, GEEE	4.92 (2.25)	4.56 (2.05)	5.37 (1.68)	*F*(2,177) = 2.48, *p* =.087

* M* =mean, *SD* standard deviation, *n* number of participants, *GEEE *Generic rating scale for previous treatment experiences, treatment expectations, and treatment effects, ranging from 0 to 10

### Outcome measures

#### Primary outcomes: Stress and depression

Stress was assessed using the *Perceived Stress Scale-10* (PSS-10, Cohen et al. 1983; Klein et al. 2016), a widely used instrument for assessing perceived stress within the last month. The time interval was adjusted to the “past two weeks”. It comprises 10 items, measuring how unpredictable, uncontrollable and/or overloaded participants felt in the past two weeks. The items have to be answered on a Likert scale from “never” (0) to “very often” (4), resulting in a score ranging from 0 to 40. Cronbach’s alpha in the original study was α = 0.78 (Klein et al. 2016).

Depressive symptoms were assessed using the depressive symptoms subscale of the *Depression Anxiety and Stress Scale 21* (DASS-21 Lovibond and Lovibond 1995), shortened and translated into German (Nilges and Essau 2015). The DASS-21 is a 21-item self-report measure of state negative affect in the past two weeks, developed with the specific aim of differentiating between depressive symptoms, anxiety and tension/stress. Each subscale comprises 7 statements to be answered on a 4-point Likert-type scale (0 = “Did not apply to me at all” —3 “Applied to me very much, or most of the time”), resulting in a sum score of 0–21 for each subscale. The German DASS-21 scale has good convergent and discriminant validity and high internal consistency (Cronbach’s α = 0.91 – depressive symptoms subscale, α = 0.82 – anxiety subscale, α = 0.89 – stress subscale (Nilges and Essau 2015).

#### Secondary outcomes: Anxiety, somatization, sleep and well-being 

Anxiety symptoms were assessed using the anxiety symptoms subscale of the *Depression Anxiety and Stress Scale 21 (DASS-21*; see primary outcomes above).

Somatization symptoms were assessed using the German version (Löwe et al. 2002) of the *Patient Health Questionnaire 15* (PHQ-15 Kroenke et al. 2002). The PHQ-15 is a 15-item, self-report measure assessing the severity of various somatic symptoms (e.g., pain, shortness of breath, feeling faint). The time interval was adjusted such that participants rated how much they had been affected by each symptom in the past two weeks (0 = “not bothered at all” to 2 = “bothered a lot”). Sum scores range from 0 to 30. The German translation of the PHQ-15 has a good internal reliability of Cronbach’s α = 0.79 (Gräfe et al. 2004).

Sleep quality was assessed using the German version (Backhaus et al. 2002) of the *Pittsburgh Sleep Quality Index* (PSQI Buysse et al. 1989), which assesses sleep quality for the previous month (e.g., “during the past month, how often have you had trouble sleeping because you wake up in the middle of the night or early morning?”). For the present study, the time interval was adjusted to the past two weeks. Based on the reported frequency of sleep problems, each of the 19 items is weighted on a 0 (positive extreme) to 3 (negative extreme) interval scale. The global PSQI score is calculated by totaling seven sleep components, providing a range from 0 (very good sleep) to 21 (very bad sleep). The PSQI is widely used in insomnia research (Buysse et al. 2006); the German version also has high reliability and validity with a high internal consistency of Cronbach’s α = 0.85 (Backhaus et al. 2002).

Mental well-being was assessed using the German version (Bachinger and Lang 2013) of the *Warwick–Edinburgh Mental Wellbeing Scale (WEMWBS* Tennant et al. 2007). This consists of 14 positively worded statements covering subjective well-being and psychological functioning within the last two weeks. The scale is scored by summing up the response to each item answered on a Likert scale from 1 (none of the time) to 5 (all of the time), thus ranging from 14 to 70. The German version of the WEMWBS has demonstrated high reliability and validity with an internal consistency of α = 0.92 (Lang and Bachinger 2017).

#### Previous treatment experiences and treatment expectations (exploratory outcomes)

Prior treatment experiences, treatment expectations and perceived improvement were assessed with the *Generic rating scale for previous treatment experiences*,* treatment expectations*,* and treatment effects (GEEE* (Rief et al. 2021)). In addition to patients’ treatment-related expectations (“How much improvement do you expect from the treatment”), the GEEE assesses both prior treatment experiences (“How much improvement have you experienced with the treatment in the past?”) and current experiences of treatment-related effects (“How much improvement have you experienced since your participation in this study?”). Importantly, treatment expectations, as well as prior and current experiences and potential side effects, are assessed using 0–10 Numeric Rating Scales (NRS). The GEEE is currently under further evaluation in a series of different studies.

### CBD intervention

We used the sublingual application of an over-the-counter 10% full-spectrum CBD-oil consisting of 90% MCT oil (carrier oil) and 10% full spectrum hemp extract (1000 mg CBD in 10 ml) with terpenes and other cannabinoids, including small amounts of THC instead of an isolate (pure CBD, no other cannabinoids or terpenes) or broad-spectrum (CBD with terpenes and other cannabinoids, but not THC) oil to mimic a naturalistic CBD user setting. For the placebo oil we used pure hemp seed oil (0 mg CBD in 10 ml).

**Fig. 2:**
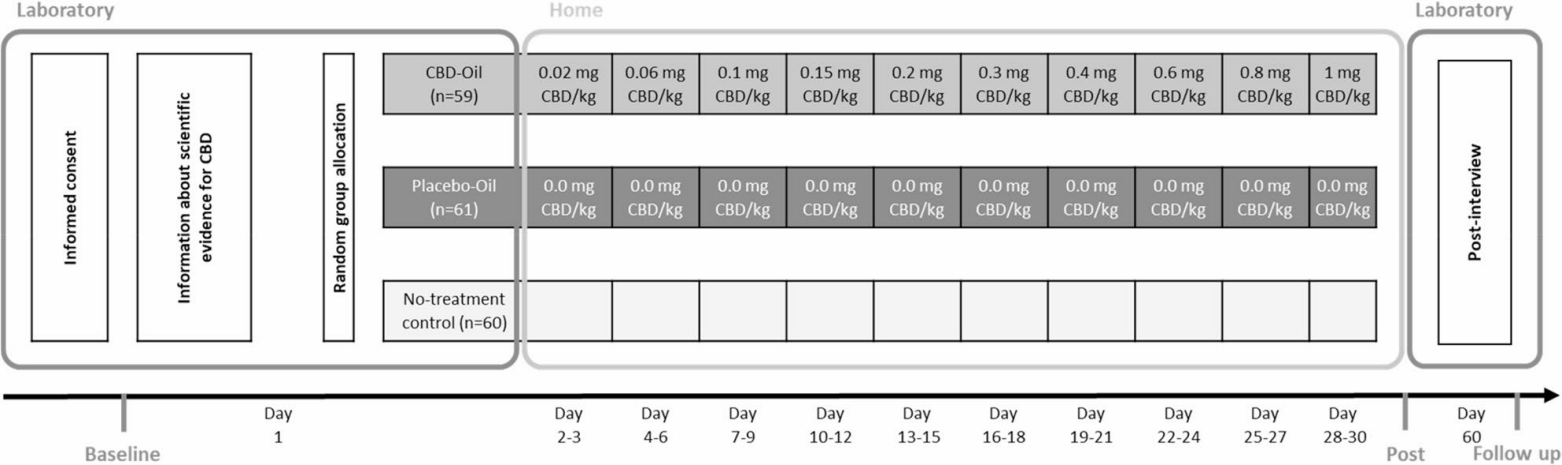

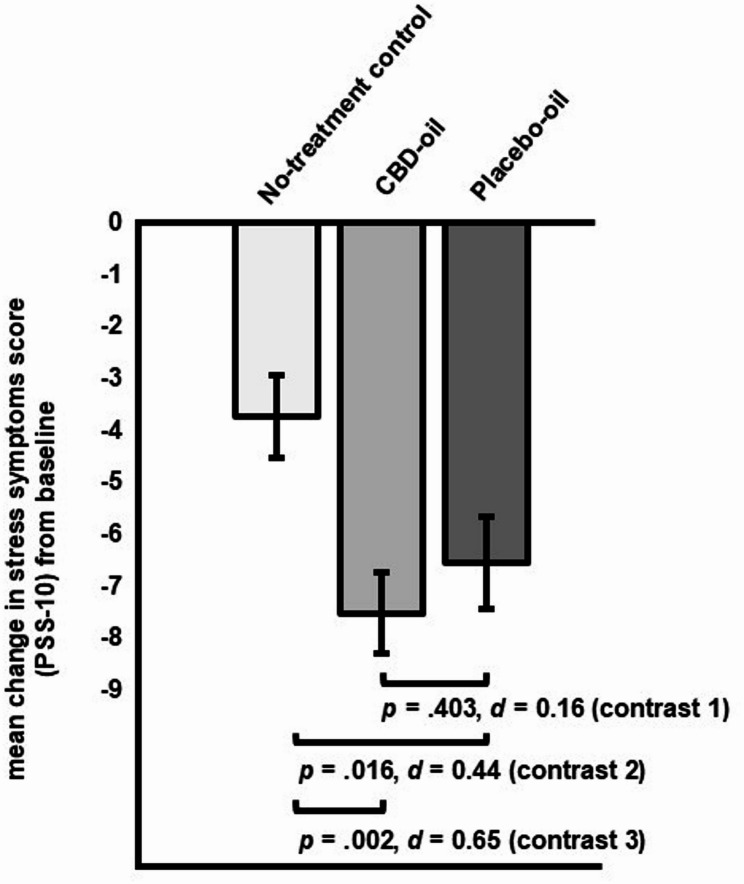
Experimental design

The dosing scheme started with 0,02 mg CBD/kg body weight at day one and the dose gradually increased every 3 days up to 1 mg CBD/kg body weight at day 30 (see Fig. 2). This scheme was based on dosing recommendations described by Leinow and Birnbaum (2019), typically recommended in the over the counter CBD user community (e.g. https://cbd360.de). For these low dosages (1,7 mg to 85 mg for a participant with 85 kg of body weight), there is so far no empirical evidence for the efficacy of CBD products in the treatment of stress and psychological distress. Since it is precisely these low doses that are frequently used in the treatment of stress and psychological distress, it is particularly important to confirm the efficacy of such doses in randomized controlled trials.

In a review of studies addressing dose-dependent efficacy and safety of CBD treatment across a diversity of health conditions (Arnold et al. 2023), virtually no significant effects across all outcomes were found in the CBD dosage range of less than 100 mg/day. Therapeutic benefits of CBD became more evident at doses greater than or equal to 300 mg/day. Arnold et al. (2023) concluded, that larger and more robust clinical trials are needed to confirm the therapeutic potential of lower (i.e., < 300 mg/day) oral doses of CBD, since “low-dose” CBD products are widely available in many countries and advertised for a diversity of health conditions without empirical evidence.

Studies have indicated that CBD is generally safe and well tolerated with relatively low potential for toxicity (WHO Expert Committee on Drug Dependence 2018). In case of severe overdose (which was not envisaged in the study), there may be potential for drug interactions, hepatic abnormalities, gastrointestinal symptoms, drowsiness, fatigue, vomiting, and somnolence (Huestis et al. 2019). The dosage of CBD used in the study (maximum 85 mg per day at a body weight of 85 kg) falls far below dosages used in studies for the treatment of epilepsy and psychiatric disorders (up to 1000 mg CBD per day) for which such side effect profile have been reported. The World Health Organization has reported that, for pure CBD, there is no evidence for misuse, abuse, or dependence (WHO Expert Committee on Drug Dependence 2018).

To avoid drug-drug interactions, participants taking regular medications, which could potentially interact with CBD oil, and participants with a known allergy to the oils’ ingredients were excluded from study participation.

### Study design

A randomized parallel group experimental design was used with participants randomly assigned to one of the three groups (CBD-oil, placebo-oil, no-treatment control). Block randomization with a block size of 30 participants was conducted by the principal investigator prior to the laboratory session to allocate participants equally to all groups using a random number generator (random.org). Experimenters, who enrolled and assigned participants were not blind to group allocation (oil vs. no-treatment control), but blinded to CBD vs. placebo-oil.

After giving informed consent, participants were asked to fill out the baseline measures using an online survey platform (Unipark; Questback, 2018). Next, all participants received the same highly standardized information about the scientific evidence for the efficacy of CBD treatment of psychological distress. Participants were informed that the scientific evidence regarding the efficacy of CBD in treating psychological distress is primarily based on basic research findings and more evidence in practice is needed. Specifically, we pointed out that to date, there are only few randomized controlled trials (RCTs) investigating the effects of low-dose CBD on stress and well-being.

Participants were then informed about their group allocation (oil vs. no-treatment control) and participants in each of two oil groups received the oil. In the two oil groups, the investigator was wearing rubber gloves and handed the bottle of oil to the participant. The participants were provided with detailed instructions for its use, and the investigator supervised the first sublingual administration. Participants were then instructed to self-administer the oil at home over the subsequent 29 days, following their individually determined dosing schedule. To enhance adherence, participants were also asked to fill out a paper and pencil protocol (including the day and time of oil intake). Participants in the no-treatment control group underwent the same procedure (informed consent, information about scientific evidence for CBD, questionnaires for baseline, post and follow-up measurement) without receiving oil treatment. After 30 days, all participants were invited via email to fill out the post-intervention measures online on study day 30, (see Fig. 2).

### Study procedure

The investigators contacted interested participants by telephone to check the inclusion and exclusion criteria and to arrange an appointment on site. The subjects first received the subject information in the laboratory, which entailed full information about the procedure and the objectives of the study. Subsequently, there was the possibility for further questions. Those who wished to participate received the consent form, which provided information about withdrawal options, compensation for expenses and data security, as well as the European Data Protection Regulation (DGSVO) to sign. Subsequently, subjects completed psychometrically validated questionnaires on stress, sleep quality, somatic symptoms, well-being, emotionality, as well as some questions on demography, experiences with CBD, and expectations regarding CBD on a PC for about 15 min. Subjects were informed by the investigator of the a priori group assignment, i.e. control group without oil or double-blind placebo oil/CBD oil. Subjects in all groups then received detailed information about the state of scientific evidence on the efficacy of CBD oil using an interactive conversation with the investigator. In total, the information transfer took 10–15 min. This was followed by the first application of CBD oil or placebo oil (depending on the group) in the presence of the experimenter with instructions on how to apply it (in the oil groups) or no application of an oil (no-treatment control group). The subjects of the oil groups were then instructed to follow the intake regimen of body weight-dependent amounts of CBD oil. Specifically, subjects received their individual dosage schedule, and were instructed to document the time and the number of drops actually taken in this sheet (see supplementary materials). Only subjects in the oil groups were given a vial of oil (10 ml). Participants in all groups were informed that they had to complete a questionnaire package on the 30th day of participation, taking approximately 15 min to fill out. At this point, in addition to the above-mentioned outcome variables, possible side effects, motivation to participate, and compliance were queried. The link to the online questionnaire was sent to the participants by e-mail. In addition, an appointment was made at the laboratory in the 3 days following the 60th day of participation, at which the subjects would be given a vial of 10% full-spectrum CBD oil worth €60 as compensation for their participation.

Participation in the study, including all laboratory appointments and the completion of all online questionnaires, took about 110 min in total.

### Statistical analysis

Statistical analyses were performed with IBM SPSS Statistics 28.0 for Windows (IBM, Armonk, NY/USA). The significance level was set at α = 0.05 and tests were two-tailed. Differences in baseline sample characteristics, prior treatment experiences, treatment expectation at baseline and perceived improvement post-intervention were analyzed using univariate analysis of variance (ANOVA) and χ²-tests. To assess group differences in primary and secondary outcomes, a 3 × 2 mixed ANOVA with group as between-factor (CBD-oil, placebo-oil, no-treatment control) and time as within-factor (pre- vs. post- treatment) was conducted. A significant group x time interaction in the ANOVA was followed up by planned contrasts comparing CBD-oil vs. placebo-oil (contrast 1), placebo-oil versus no-treatment control (contrast 2) and CBD-oil versus no-treatment control (contrast 3). In addition, a sensitivity analysis was done to determine the minimum detectable effects size for these post-hoc contrasts which yielded an effect size of *d* = 0.48 (80% power). Since less than 10% of participants did not complete the post-treatment assessment, an intent-to-treat analysis was not considered necessary. Additionally, the Reliable Change Index (RCI; Jacobson and Truax 1991) as a measure of reliable change was calculated for all primary outcomes and compared between groups via χ²-tests. Cronbach’s alpha of the PSS-10 (α = 0.84) and the depressive symptoms scale of the DASS-21 (α = 0.91) was used as reliability coefficient to calculate RCI. Finally, we conducted a blinding sensitivity analysis comparing the two oil-groups. There was no significant group difference with regard to the number of participants believing that the oil had actually contained CBD (CBD group: 44.9%; placebo group: 42.9%; χ² (1) = 0.044, *p* =.846).

## Results

### CBD effect on stress and depressive symptoms as primary outcomes

The 3 × 2 ANOVA yielded a significant interaction effect group x time (*F*[2, 163] = 5.56, *p* =.005, partial *η*² = 0.064, see Table 2), for stress symptoms. Overall, stress symptoms decreased from pre- to post-intervention (main effect time: *F*[1, 163] = 154.02, *p* <.001, partial *η*² = 0.486). Groups did not differ significantly (main effect group: *F*[2, 163] = 2.14, *p* =.121, partial *η*² = 0.026). In terms of depressive symptoms, the 3 × 2 ANOVA yielded a significant interaction effect group x time (*F*[2, 163] = 7.02, *p* =.001, partial *η*² = 0.079, see Table 2). Moreover, there was a significant reduction in depressive symptoms (main effect time: *F*[1, 163] = 79.67, *p* <.001, partial *η*² = 0.328). There was no significant main effect of group (*F*[2, 163] = 0.335, *p* =.716, partial *η*² = 0.004).

**Table 2:** Primary and secondary outcomes at baseline and post-intervention per group, main effects (group, time), and group x time interaction

	CBD-oil (*n* = 52)				Placebo-oil (*n* = 58)				No-treatment control (*n* = 56)				Main effect group		Main effect time		Group x time interaction	
	baseline assessment		Post assessment		baseline assessment		Post assessment		baseline assessment		Post assessment							
	*M* *(SD)*	*SE*	*M* *(SD)*	*SE*	*M* *(SD)*	*SE*	*M* *(SD)*	*SE*	*M* *(SD)*	*SE*	*M* *(SD)*	*SE*	*F* *(2*,*163)*	*p*	*F* *(1*,*163)*	*p*	*F* *(2*,*163)*	*p*
Primary outcomes																		
Stress (PSS-10)	26.25 (4.43)	0.61	18.71 (5.50)	0.76	24.67 (4.57)	0.60	18.12 (5.52)	0.73	24.80 (4.13)	0.55	21.05 (6.16)	0.82	2.14	0.121	154.02	< 0.001	5.56	0.005
Depression (DASS-21)	8.67 (5.04)	0.70	4.77 (4.21)	0.58	7.98 (4.18)	0.55	4.31 (3.42)	0.45	7.05 (3.99)	0.53	5.86 (4.54)	0.61	0.34	0.716	79.67	< 0.001	7.02	0.001
Secondary outcomes																		
Anxiety (DASS-21)	6.90 (4.48)	0.62	3.67 (3.96)	0.55	5.14 (3.49)	0.46	2.97 (3.16)	0.42	5.79 (3.93)	0.53	4.00 (3.45)	0.46	2.03	0.134	75.16	< 0.001	2.37	0.097
Somatization (PHQ-15)	11.25 (5.12)	0.71	7.12 (3.87)	0.54	10.67 (4.41)	0.58	6.55 (4.46)	0.59	11.46 (4.11)	0.55	9.20 (3.68)	0.49	3.00	0.053	127.43	< 0.001	4.01	0.020
Sleep Quality (PSQI)	9.02 (2.88)	0.40	6.91 (3.36)	0.47	7.82 (3.15)	0.41	6.28 (3.07)	0.40	7.74 (3.53)	0.47	6.94 (2.63)	0.35	1.42	0.245	36.74	< 0.001	2.43	0.091
Well-being, (WEMWBS)	39.65 (7.73)	1.07	44.33 (7.26)	1.01	42.17 (6.55)	0.86	46.69 (6.44)	0.85	41.59 (6.10)	0.82	44.14 (7.51)	1.00	2.47	0.088	46.17	< 0.001	1.41	0.248

*M* mean, *SD* standard deviation, *SE* standard error, *n* number of participants, *PSS-10 * Perceived Stress Scale (ranging from 0 to 40), *DASS-21* Depression Anxiety and Stress Scale (each scale ranging from 0 to 21), PHQ-15 Somatization scale from the Patient Health Questionnaire (ranging from 0 to 30), *PSQI* Pittsburgh Sleep Quality Index (ranging from 0 to 21), *WEMWBS* Warwick–Edinburgh Mental Wellbeing Scale (ranging from 14 to 70)

**Fig. 3:**
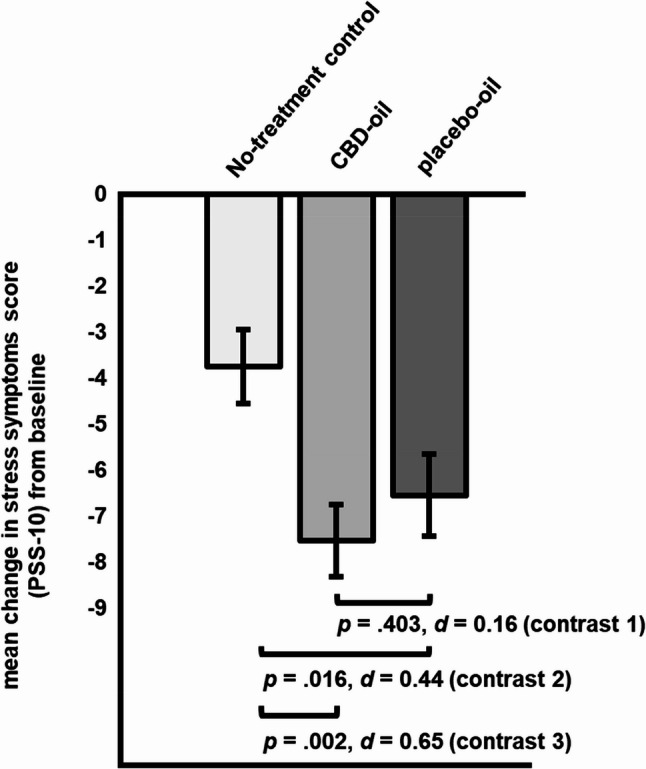

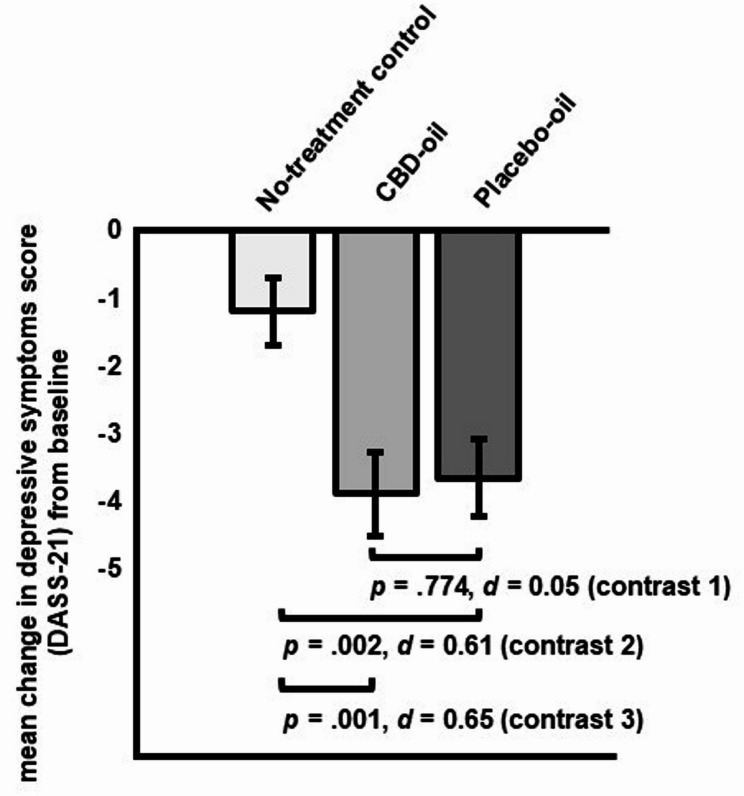
Planned contrasts for change in depressive symptoms (*M*, *SE*; DASS-21) from pre- to post-intervention for all groups (contrast 1: CBD-oil vs. placebo-oil; contrast 2: placebo-oil vs. no-treatment control; contrast 3: CBD-oil vs. no-treatment control)

Planned contrasts comparing CBD-oil and placebo-oil (contrast 1) yielded a non-significant difference in stress change scores (∆ _post – pre intervention_ = 0.99, *SE* = 1.18, *p* =.403, *d* = 0.16 [95% CI, −0.21 to 0.54]; see Fig. 3). With regard to changes in perceived stress, the placebo-oil (contrast 2: ∆ _post – pre intervention_ = −2.80, *SE* = 1.16, *p* =.016, *d* = 0.44 [95% CI, 0.07 to 0.81]) and the CBD oil group (contrast 3: ∆ _post – pre intervention_ = −3.79, *SE* = 1.19, *p* =.002, *d* = 0.65 [95% CI, 0.26 to 1.04]) reported significantly greater changes when compared to the no-treatment control group (within group effect sizes: CBD: *d* = 1.51; placebo oil: *d* = 1.37).

**Fig. 4:**
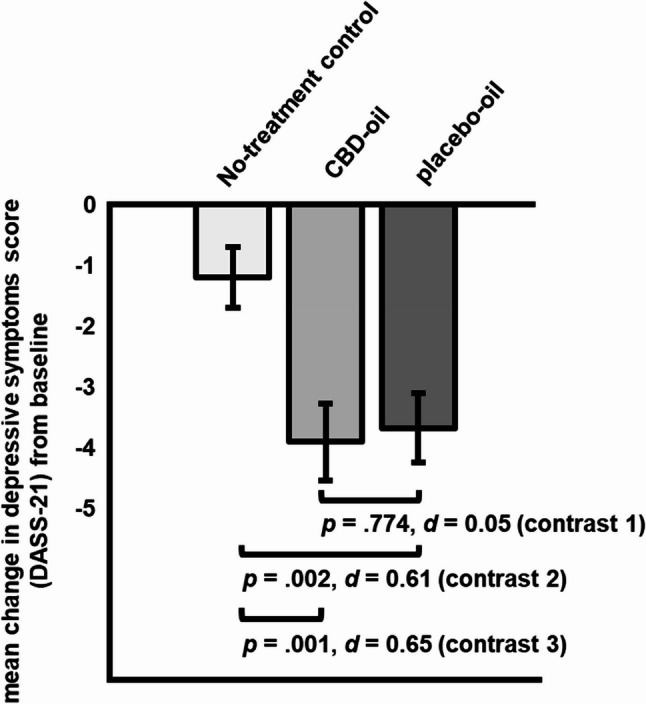

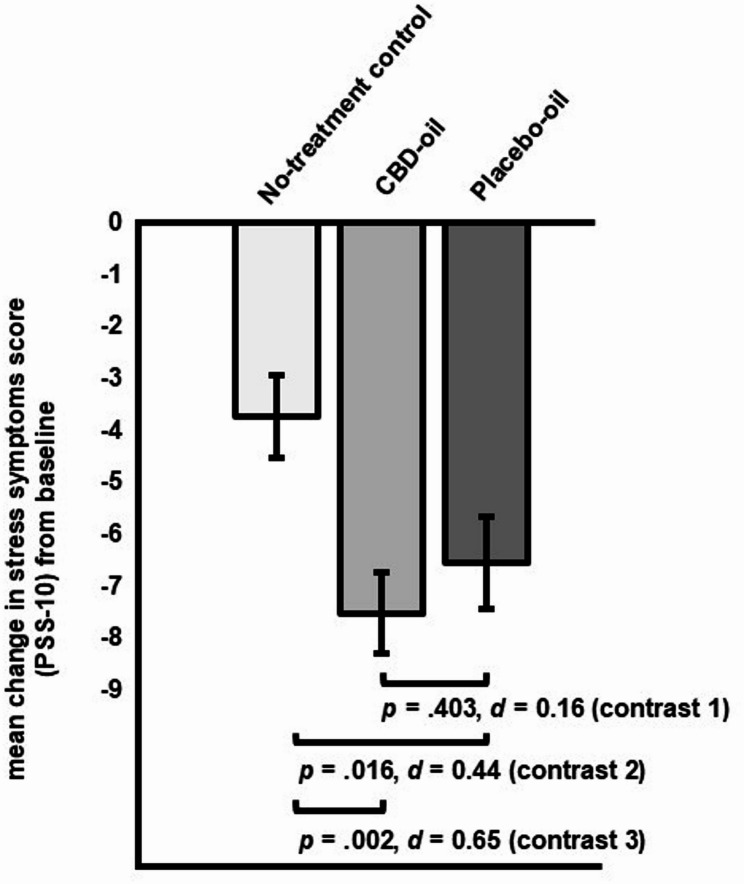
Planned contrasts for change in stress symptoms (*M*, *SE*; PSS-10) from pre- to post-intervention for all groups (contrast 1: CBD-oil vs. placebo-oil; contrast 2: placebo-oil vs. no-treatment control; contrast 3: CBD-oil vs. no-treatment control)

Planned contrasts comparing CBD-oil vs. placebo-oil (contrast 1) yielded a non-significant difference in depressive symptoms change scores (∆ _post – pre intervention_= 0.23, *SE* = 0.81, *p* =.774, *d* = 0.05 [95% CI, −0.32 to 0.42]; see Fig. 4). Depressive symptoms decreased significantly more in the placebo-oil (contrast 2: ∆ _post – pre intervention_= −2.48, *SE* = 0.79, *p* =.002, *d* = 0.61 [95% CI, 0.23 to 0.99]) and the CBD-oil group as compared to the no-treatment control group (contrast 3: ∆ _post – pre intervention_= −2.71, *SE* = 0.81, *p* =.001, *d* = 0.65 [95% CI, 0.26 to 1.04]; within group effect sizes: CBD: *d* = 0.81; placebo oil: *d* = 1.04).

*Reliable change.* The proportion of participants within each group with a Reliable Change Index (RCI) > 1.96 is summarized in Table 3. There was a significant difference between groups (χ²(2) = 7.24, *p* =.027, φ = 0.21) concerning the number of participants with a reliable reduction in stress symptoms with the CBD and the placebo group not differing significantly (χ²(1) = 0.27, *p* =.603) and with the CBD (χ²(1) = 6.31, *p* =.012) and the placebo (χ²(1) = 4.26, *p* =.039) group yielding a higher number of participants with a reliable change in stress symptoms compared to no-treatment control. For the number of participants with a reliable reduction in depressive symptoms, there was a significant difference between groups (χ²(2) = 8.21, *p* =.016, φ = 0.22), with the CBD and the placebo group not differing significantly (χ²(1) = 0.39, *p* =.530) and with the CBD (χ²(1) = 4.49, *p* =.034) and the placebo (χ²(1) = 7.77, *p* =.005) group yielding a higher number of participants with a reliable change in depressive symptoms compared to no-treatment control.

**Table 3:** Number of participants with a reliable change index (RCI) > 1.96 as a measure of reliable change in stress symptoms and depressive symptoms per group

	CBD-oil (*n* = 52)			Placebo-oil (*n* = 58)			No-treatment control (*n* = 56)	
	*n*	%		*n*	%		*n*	%
Stress symptoms, PSS-10	33	63.5		34	58.6		22	39.3
Depressive symptoms, DASS-21	22	19.7		28	48.3		13	23.2

Note. *n* = number of participants; PSS-10 = Perceived Stress Scale; DASS-21 = Depression Anxiety and Stress Scale

### CBD effect on anxiety, somatization symptoms, sleep quality and mental well-being

The 3 × 2 ANOVAs yielded no significant interactions of group x time for anxiety symptoms (*F*[2, 163] = 2.37, *p* =.097, partial *η*² = 0.028), sleep quality (*F*[2, 163] = 2.43, *p* =.091, partial *η*² = 0.032) or mental well-being (*F*[2, 163] = 1.41, *p* =.248, partial *η*² = 0.017), as displayed in Table 2. The 3 × 2 ANOVA yielded a significant interaction of group x time for somatization symptoms (*F*[2, 163] = 4.01, *p* =.020, partial η² = 0.047).

There was a significant reduction (main effect time) for anxiety symptoms (*F*[1, 163] = 75.16, *p* <.001, partial *η*² = 0.316), somatization symptoms (*F*[1, 163] = 127.43, *p* <.001, partial *η*² = 0.439), and a significant improvement of sleep quality (*F*[1, 163] = 36.74, *p* <.001, partial *η*² = 0.199) and mental well-being (*F*[1, 163] = 46.17, *p* <.001, partial *η*² = 0.221) over time, as displayed in Table 2. For none of the secondary outcomes, a significant group main effect was obtained: anxiety symptoms (*F*[2, 163] = 2.03, *p* =.134, partial *η*² = 0.024), somatization symptoms (*F*[2, 163] = 3.00, *p* =.053, partial *η*² = 0.035), sleep quality (*F*[2, 163] = 1.42, *p* =.245, partial *η*² = 0.019), and mental well-being (*F*[2, 163] = 2.47, *p* =.088, partial *η*² = 0.029) (see Table 2).

**Fig. 5:**
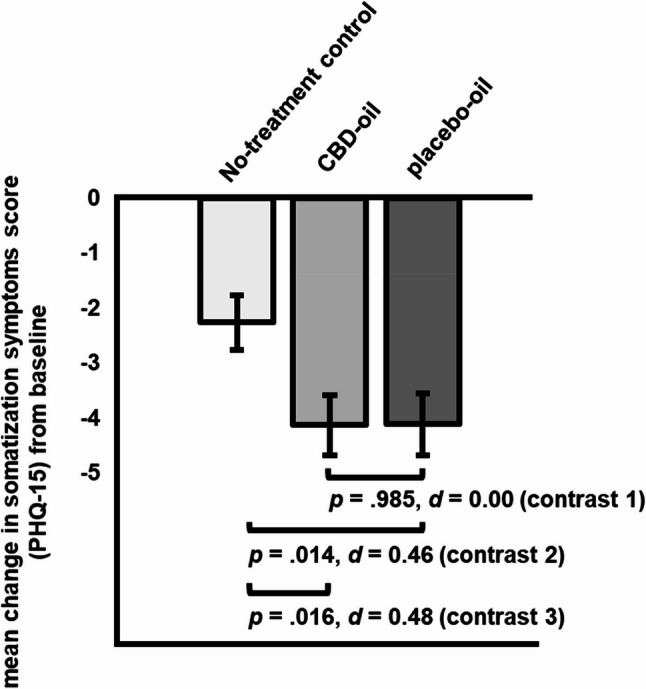

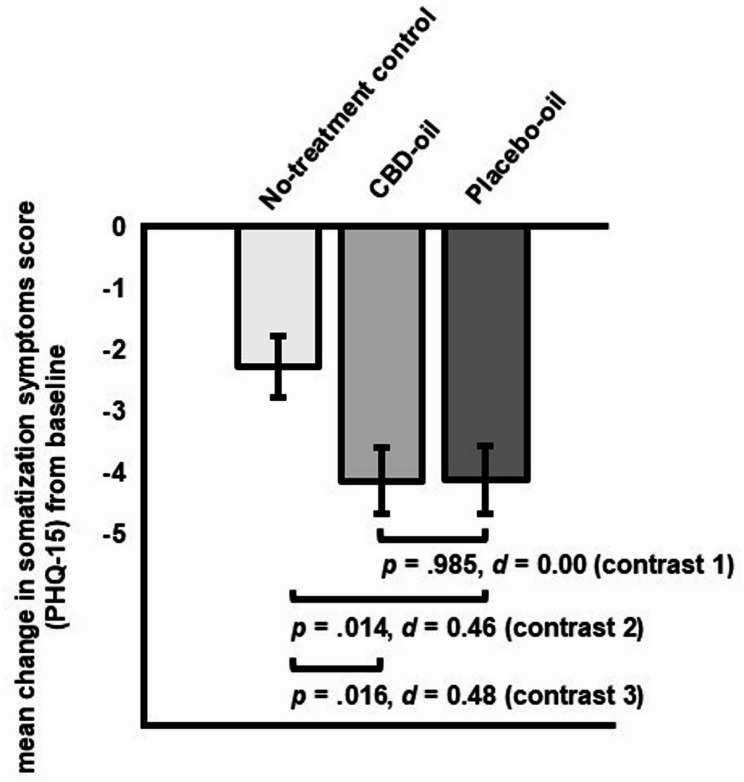
Planned contrasts for change in somatization symptoms (*M*, *SE*; PHQ-15) from pre- to post-intervention for all groups (contrast 1: CBD-oil vs. placebo-oil; contrast 2: placebo-oil vs. no-treatment control; contrast 3: CBD-oil vs. no-treatment control)

Planned contrasts comparing CBD-oil vs. placebo-oil (contrast 1) yielded a non-significant difference in somatization symptoms change scores (∆ _post – pre intervention_= 0.14, *SE* = 0.76, *p* =.985, *d* = 0.00; [95% CI, −0.37 to 0.37] see Fig. 5). Somatization symptoms decreased significantly more in the placebo-oil (contrast 2: ∆ _post – pre intervention_= −1.85, *SE* = 0.75, *p* =.014, *d* = 0.46 [95% CI, 0.09 to 0.83]) and the CBD-oil group (contrast 3: ∆ _post – pre intervention_= −1.87, *SE* = 0.77, *p* =.016, *d* = 0.48 [95% CI, 0.10 to 0.86]) as compared to the no-treatment group.

### Prior treatment experiences, treatment expectations, perceived improvement

At baseline, there were no significant group differences in prior treatment experiences (*F*[2, 77] = 0.47, *p* =.630) and treatment expectations (*F*(2,177) = 2.48, *p* =.087). Moreover, after treatment, groups (CBD-oil group *M* = 3.08, *SD* = 2.65; placebo-oil group *M* = 3.39, *SD* = 2.81) did not differ (*F*[1, 104] = 0.37, *p* =.547) concerning perceived improvement (0 = no improvement to 10 = greatest improvement imaginable).

Within the placebo-oil group, change in stress symptoms (*r* = −.26, *p* =.052), depressive symptoms (*r* =.05, *p* =.687), anxiety symptoms (*r* =.06, *p* =.645), somatization symptoms (*r* =.04, *p* =.754), sleep (*r* = −.13, *p* =.355) and wellbeing (*r* =.22, *p* =.105) were not correlated with treatment expectation. Interestingly, the direct rating of treatment efficacy concerning positive mood and mental health (*r* =.36, *p* =.006), pain relief (*r* =.37, *p* =.005), harm reduction (*r* =.36, *p* =.006), sleep (*r* =.34, *p* =.010) and perception of no effect (*r* = −.37, *p* =.005) were correlated with treatment expectation.

Within the CBD-oil group, change in stress symptoms (*r* = −.19, *p* =.174), depressive symptoms (*r* = −.02, *p* =.880), anxiety symptoms (*r* = −.11, *p* =.431), somatization symptoms (*r* = −.05, *p* =.702), sleep (*r* = −.09, *p* =.533) and wellbeing (*r* =.20, *p* =.151) were not correlated with treatment expectation. The direct rating of perceived treatment efficacy concerning positive mood and mental health (*r* =.22, *p* =.122), pain relief (*r* = −.00, *p* =.985), harm reduction (*r* =.05, *p* =.751), sleep (*r* =.02, *p* =.897) and perception of no effect (*r* =.06, *p* =.707) were also not correlated with treatment expectation.

## Discussion

Our findings indicate a substantial placebo response in a 30-day low dose CBD treatment of stress, depressive symptoms, and somatization, but not for other markers of psychological distress in highly stressed students. While participants in the CBD-oil and placebo-oil group benefitted significantly more regarding their stress (CBD: *d* = 0.65; placebo: *d* = 0.44) and depressive symptoms (CBD: *d* = 0.65; placebo: *d* = 0.61) than the no-treatment group, no significant difference in any outcome was shown between the CBD-oil and the placebo-oil group (stress: *d* = 0.16; depressive symptoms: *d* = 0.05).

The effects in the CBD-oil group compared to the no-treatment control group are in line with recent reviews summarizing the findings on the effect of CBD in psychiatric disorders (Kirkland et al. 2022; Bonaccorso et al. 2019). For example, in a study (Masataka 2019) comparing 300 mg CBD for 4 weeks vs. placebo in Japanese adolescents with social anxiety disorder, CBD significantly reduced anxiety symptoms. In a very small placebo-controlled trial, a single dose of 400 mg CBD (Crippa et al. 2011) significantly decreased subjective anxiety and altered regional cerebral blood flow in patients with social anxiety disorder. While such studies suggest CBD’s potential to reduce anxiety or emotional distress, they are difficult to compare with the present study due to their focus on clinical samples and higher CBD doses. Crippa et al.’s (2021) study is more comparable to the present study, even though it was not placebo-controlled. In this study, a 28-day CBD (300 mg/day) intervention led to a significant reduction in emotional exhaustion in healthcare workers during the COVID-19 pandemic due to CBD intake. Similarly, we observed a significant decrease of stress and depressive symptoms in the CBD group. Interestingly, the within-group effect size for emotional exhaustion ($\:{{\upeta\:}}^{2}$ at day 28 = 0.04, yielding *d* = 0.41) reported by Crippa et al. (2021) for the CBD group was lower in magnitude as compared to the within-group effect size for stress (*d* = 1.51) and depressive symptoms (*d* = 0.81) we obtained, even though the dosage in our study was much lower (e.g., 1 mg/kg/day). Most importantly, in our study, the efficacy of CBD oil was not superior to the placebo oil. This suggests, that at least the benefits of low dose CBD oil are largely accounted for by a placebo effect. This may also be true for higher CBD oil doses given that, at least in non-psychiatric samples such as in the Crippa et al. (2021) study, the observed effects sizes were not considerably higher than the one we observed.

We demonstrated for the first time in a parallel group design including a CBD group, a placebo group, and a no-treatment control group that placebo effects play a substantial role in accounting for CBD effects in the treatment of psychological distress.

While there is no other study so far assessing placebo effects in CBD treatment of stress in a controlled manner, Spinella et al. (2021) demonstrated that CBD expectancy alone altered several subjective and physiological responses, at least when taken in an experimental session. In a randomized crossover design involving 43 healthy adults, participants self-administered CBD-free hempseed oil, but were told it contained CBD in one session, and that it was CBD-free in another. While CBD expectancy was associated with increased sedation and changes in heart rate variability indicative of heightened anticipatory stress regulation, subjective stress and anxiety levels did not significantly change based on expectancy. However, participants who strongly believed in CBD’s anxiolytic properties reported reduced anxiety in the CBD expectancy condition, suggesting the importance of considering expectancy-related factors in CBD research (Spinella et al. 2021).

In a recent study (Spinella et al. 2023), CBD expectancy was shown to reduce cortisol release levels in anticipation of stress in a lab stress test, particularly in males, emphasizing the importance of considering drug-related expectations when assessing CBD’s effects on stress. Similarly, in another experimental study, CBD expectancy was associated with increased subjective sedation and tended to be associated with blunted subjective stress, replicating the findings that CBD expectancy alone can impact stress- and anxiety-relevant responses (Zhekova et al. 2024). Our results extend previous findings, as we evaluated a four-week intake of the placebo and CBD oil in a typical CBD user sample of students suffering from stress.

In order to convey the notion of a valid treatment, we used a commercially available CBD oil in its original packaging provided by the manufacturer. The placebo oil was delivered in an identical manner. Moreover, participants received an individualized dosing scheme, detailed instructions on sublingual administration, and took their first dose under supervision of the investigator, thus creating a treatment context. Our study thus provides strong evidence that the stress-reducing effects of self-treatment with sublingually applied CBD oil in the doses commonly used in the community and recommended by the manufacturers are predominantly due to placebo effects. The dosing regimen we applied started with 0.02 mg CBD/kg body weight at day one and gradually increasing every 3 days up to 1 mg CBD/kg body weight at day 30. It was based on dosing recommendations described by Leinow and Birnbaum (2019), corresponding with recommendation of many distributors of over the counter CBD products and the CBD user community and is therefore adequately for addressing the efficacy of the usual over the counter CBD product usage. For these low dosages (under 100 mg/day), there is so far no empirical evidence for the efficacy of CBD products, in the treatment of stress and psychological distress. Nonetheless, the obtained within-group effects sizes for the reduction of stress and depressive symptoms were at least as high to those observed by Crippa et al. (2021) in a sample of health workers treated with a high CBD dosage. As the dosing regimen we used is a common one in the community of CBD users, it is of particular importance to close this research gap and provide empirical evidence.

We also explored the role of treatment expectation as the underlying mechanism of the placebo response. Within both oil group change in all outcomes was not correlated with treatment expectation. Interestingly, exclusively within the placebo-oil group, the direct rating of perceived treatment efficacy was correlated with treatment expectation, indicating that treatment expectation plays primarily a role in placebo effects if the outcome is directly assessed, a result that has already been found in several fields of research in which placebo effects have been investigated (e.g. Winkler and Hermann 2019).

Some limitations should be noted. Our sample of highly stressed female dominant university students is rather selective concerning gender, educational level and age. Since elevated psychological distress as a university student predicts an increase in likelihood of mental disorders later in life (Woo et al. 2024),we included an at-risk sample and determined reliable change, thus allowing clinical implications. The selection of a highly stressed population might induced regression to the mean effects that might overshadow treatment effects of the oil conditions. To address this methodological bias, we used a randomized controlled parallel group design including a no-treatment control group. By comparing change in oil conditions against change in the no-treatment control group, we controlled for regression to the mean effects. Beyond that, we did not assess participants’ health-related behavior change, which might account for the observed benefit in the oil groups. Another issue might be insufficient power. A post-hoc power analysis revealed that given the observed between-group effect sizes (stress: *d* = 0.16; depressive symptoms: *d* = 0.05) and the sample sizes, the study had a power of 21% (stress) and 8% (depressive symptoms) to detect a statistically significant effect. While this implies a considerably Type II error, it is highly questionable whether such small effect size for the difference between CBD and the placebo group would be clinically meaningful. Moreover, we conducted an a priori power analysis, thus ascertaining adequate power for an assumed small effects size of the group x time interaction.

The route of administration (ROA) plays a critical role in the pharmacokinetics and therapeutic outcomes of CBD. Similar to previous studies (e.g. Crippa et al. 2021; Linares et al. 2019; Masataka 2019) and the typical ROA of commercially available CBD, in the present study, CBD was administered sublingually, a method that allows for partial absorption through the oral mucosa directly into the systemic circulation, thereby bypassing first-pass hepatic metabolism (Millar et al. 2018). Compared to oral ingestion, which often leads to delayed onset and reduced bioavailability due to extensive first-pass metabolism, sublingual administration may result in more rapid and reliable systemic exposure (Zgair et al. 2016). Other common ROAs include inhalation, with rapid onset of CBD effects, which might have produced different results although it may not be suitable for all populations. A final ROA is topical/transdermal application, which induces localized effects and is associated with limited systemic absorption (Bruni et al. 2018). Further research is needed to systematically examine how variations in ROA impact clinical outcomes, especially in relation to dosage, onset time, and bioavailability.

Another possible explanation for the lack of efficacy might be the low dosage, possibly below the therapeutic threshold. Since “low-dose” CBD products are widely available in many countries and advertised for a variety of health conditions without empirical evidence, it is crucial to have more placebo-controlled studies determining the optimal therapeutic doses of CBD for translating first evidence of predominantly experimental research findings into clinical practice. Future studies should assess the therapeutic potential of higher sublingual doses of CBD in the treatment of stress and psychosocial distress in (sub-) clinical samples. Moreover, future studies should focus on affective symptoms, since so far no clinical trials investigating the efficacy of CBD assessed affective symptoms as the primary outcome (Pinto et al. 2020). Many important questions, such as the long-term effects and mechanisms of action (like the role of the placebo effect and treatment expectation), remain unanswered, highlighting the need for further research.

## Conclusions

In summary, our findings demonstrate that the treatment effects of sublingual low-dose CBD oil on perceived stress and psychosocial distress are predominantly accounted for by placebo effects. The popularity of such over-the-counter CBD products can be explained by moderate effect sizes in the reduction of stress, depression and somatization symptoms between the CBD-oil and no-treatment control group. While a priori treatment expectation does not account for the obtained improvements, it correlates with the subjectively perceived effectiveness at post treatment. In turn, this might strengthen treatment expectations and motivate for continuing CBD use.

## Supplementary Information

Supplementary material 1

## Data Availability

The dataset supporting the conclusions of this article is available in the zenodo open-access repository, [10.5281/zenodo.17681223].
